# Alcohol and the risk of all-cause death, atrial fibrillation, ventricular arrhythmia, and sudden cardiac arrest

**DOI:** 10.1038/s41598-024-55434-6

**Published:** 2024-02-29

**Authors:** Yun Gi Kim, Dong Yun Kim, Seung-Young Roh, Joo Hee Jeong, Hyoung Seok Lee, Kyongjin Min, Yun Young Choi, Kyung-Do Han, Jaemin Shim, Jong-Il Choi, Young-Hoon Kim

**Affiliations:** 1 Division of Cardiology, Department of Internal Medicine, Korea University College of Medicine and Korea University Anam Hospital, 73 Goryeodae-ro, Seongbuk-gu, Seoul, 02841 Republic of Korea; 2 Korea University College of Medicine, Seoul, Republic of Korea; 3 Division of Cardiology, Department of Internal Medicine, Korea University College of Medicine and Korea University Guro Hospital, Seoul, Republic of Korea; 4 Department of Statistics and Actuarial Science, Soongsil University, Seoul, Republic of Korea

**Keywords:** Sudden cardiac arrest, Atrial fibrillation, Ventricular arrhythmia, Alcohol, Cardiology, Risk factors

## Abstract

The risk of having atrial fibrillation (AF) is associated with alcohol intake. However, it is not clear whether sudden cardiac arrest (SCA) and ventricular arrhythmia (VA) including ventricular tachycardia, flutter, or fibrillation have similar associations with alcohol. We aimed to evaluate the association of alcohol intake with all-cause death, new-onset AF, VA, and SCA using single cohort with a sufficient sample size. A total of 3,990,373 people without a prior history of AF, VAs, or SCA was enrolled in this study based on nationwide health check-up in 2009. We classified the participants into four groups according to weekly alcohol consumption, and evaluated the association of alcohol consumption with each outcome. We observed a significant association between mild (hazard ratio [HR] = 0.826; 95% confidence interval [CI] = 0.815–0.838) to moderate (HR = 0.930; 95% CI = 0.912–0.947) drinking with decreased risk of all-cause mortality. However heavy drinking (HR = 1.108; 95% CI = 1.087–1.129) was associated with increased all-cause death. The risk of new-onset AF was significantly associated with moderate (HR = 1.129; 95% CI = 1.097–1.161) and heavy (HR = 1.298; 95% CI = 1.261–1.337) drinking. However, the risk of SCA showed negative association with all degrees of alcohol intake: 20% (HR = 0.803; 95% CI = 0.769–0.839), 15% (HR = 0.853; 95% CI = 0.806–0.902), and 8% (HR = 0.918; 95% CI = 0.866–0.974) lower risk for mild, moderate, and heavy drinkers, respectively. Mild drinking was associated with reduced risk of VA with moderate and heavy drinking having no associations. In conclusion, the association between alcohol and various outcomes in this study were heterogeneous. Alcohol might have different influences on various cardiac disorders.

## Introduction

Sudden cardiac arrest (SCA) is one of the leading causes of cardiovascular mortality worldwide, accounting for approximately half of all deaths from cardiovascular disease^1^. The majority of people with SCA had no previous history of heart disease making it a challenging task to assess the risk or establish strategies for prevention^2,3^.

According to previous studies, various factors such as hypertension, diabetes mellitus, or metabolic syndrome are known to increase the risk for SCA^1,4–7^. Ventricular arrhythmias (VA) such as ventricular tachycardia (VT), ventricular flutter (VFL), or ventricular fibrillation (VF) are the main cause of SCA and share similar risk factors^2,8^. The impact of alcohol on VA and SCA is controversial. A prior prospective cohort study of 7,735 men showed that heavy drinking is associated with a two-fold increased risk of sudden cardiac death^9^. In contrast, Albert et al. reported that men who consumed low-to-moderate alcohol (2–6 drinks per week) had a significantly lower risk of sudden cardiac death compared with rare-to-never drinkers^10^. In that study, the risk of sudden cardiac death was not increased in people who drank daily^10^. A recent observational study of 408,712 individuals showed that total alcohol consumption has a U-shaped association with SCA but not with VA^11^.

Even though ‘low-to-moderate’ consumption of alcohol is associated with lower cardiovascular mortality, alcohol can have various toxic effects on the heart^12^. Long-term heavy alcohol consumption can cause increased left ventricular mass, dilation of the ventricles, thinning of walls, and reduced contractility, which is known as alcoholic cardiomyopathy^13^. Atrial cardiomyocytes are also subject to the toxic effects of alcohol, and particularly heavy or frequent alcohol consumption is considered a strong risk factor for atrial fibrillation (AF)^14,15^. It is not clear why alcohol has a different influence on AF vs. VA.

We performed this nationwide population-based analysis to (i) determine the impact of alcohol on VA and SCA and (ii) compare the influence of alcohol on AF vs. VA and SCA.

## Methods

### Study participants

This study used the database from the National Health Insurance Service of the Republic of Korea (K-NHIS). This database can represent the entire population of the Republic of Korea since all citizens are mandatory subscribers to the service. The K-NHIS offers a regular health screening to all subscribers and accumulates the results in the database. Therefore, the database comprises various health-related information collected from nationwide health check-ups; for example, a health questionnaire about alcohol consumption habits, smoking status, and exercise level; medical measurements for body weight, height, and blood pressure; results of laboratory tests such as fasting blood glucose or lipid profiles. The K-NHIS database contains the reports of International Classification of Disease, 10th edition (ICD-10) diagnostic codes of AF, VT, VFL, VF, SCA, and other medical conditions throughout the entire nation. Mortality data is also stored in the K-NHIS database. Since subscription fee is determined by the income of the subscriber, the K-NHIS has data on income level of its subscribers. Due to the mandatory subscription, it offers data with a sufficient duration of follow-up, while being free of follow-up losses except immigrations.

This study included patients who received a nationwide health screening in 2009. The screening period was set from January 2002 to December 2008 to identify baseline medical history of the included patients. The endpoint of the study was a new onset of AF, VA (VT, VFL, VF), or SCA. Patients were excluded if they were (1) aged less than 20 years at the 2009 health screening; (2) previously diagnosed with AF, VA, or SCA during the screening period; or (3) had missing data on alcohol consumption. The current study and the protocol were approved by the official review committee of the K-NHIS and the institutional review board of Korea University Medicine Anam Hospital. Written informed consent was waived by institutional review board of Korea University Medicine Anam Hospital due to the retrospective nature of the study. The legal regulations of the Republic of Korea and the ethical guidelines of the 2013 Declaration of Helsinki were strictly followed.

### Alcohol intake

This study uses two parameters included in the questionnaire of the national health screening: the number of days per week a respondent drinks and the amount a respondent consumes in a drinking session. We defined a standardized cup as a different volume for each type of alcoholic beverage but can hold a similar amount of alcohol (ethanol). For example, a 220 mL-sized standard cup for beer and a 50 mL-sized cup for Soju (a traditional Korean alcohol) can be calculated to hold about 8 g of alcohol each^15^. Based on the exact amount of alcohol intake measured, the total amounts of alcohol consumed per week were calculated, and participants were classified into four groups: (i) non-drinkers: 0 g per week; (ii) mild drinkers: less than 105 g per week; (iii) moderate drinkers: less than 210 g per week; (iv) heavy drinkers: equal to or more than 210 g per week (Supplementary Table S1).

### Definitions

The diagnoses of AF, VA (VT, VFL, VF), and SCA were based on the record of International Classification of Disease, 10th revision codes (ICD-10) in the K-NHIS database. The exact ICD-10 diagnostic codes for diseases related to this study are described in Supplementary Table S2. The strategy of ICD-10 code-based diagnosis is validated in our multiple publications^6,7,16,17^. The incidence of new-onset AF, VT, VFL, VF, or SCA was calculated as the number of newly diagnosed cases of each disease per 1,000 patient-years of follow-up. Smoking habit, diabetes mellitus, hypertension, and dyslipidemia were classified into three groups in this study, which is described in Supplementary Table S3. The status of regular physical activity was evaluated according to the self-questionnaire during the 2009 K-NHIS checkup. People who had one or more sessions in a week with moderate- or high-intensity activity were considered as having regular exercise. Chronic kidney disease (CKD) was defined as estimated glomerular filtration rate (eGFR) less than 60 mL/min/1.73m^2^, calculated by the Modification of Diet in Renal Disease (MDRD) equation. Thyroid disease, liver cirrhosis, and cancer were defined based on relevant ICD-10 codes. Heart failure was also based on ICD-10 codes but only those reported during inpatient admission were counted. The robustness of the definitions stated above has been validated in our prior publications^6,7,16,17^.

### Statistical analysis

Comparison between continuous variables was performed by Student’s *t*-test and ANOVA, the results of which are presented as mean ± standard deviation. Chi-square test was used to make a comparison between categorical values, and the results are described as percentile values. The hazard ratio (HR) and 95% confidence interval (CI) were analyzed by multivariate Cox regression analysis. Multivariate model 1 is adjusted for age and sex; model 2 for model 1 plus body-mass index (BMI), smoking status, regular exercise habit, and income level; model 3 for model 2 plus hypertension, diabetes mellitus, dyslipidemia, and CKD; and model 4 for model 3 plus history of heart failure and thyroid disease. Covariates that showed significance difference among groups stratified by alcohol consumption amount were included in the model. Variables that were associated with the risk of SCA in our prior work was also included in the model^18^. For all multivariate adjustments, non-drinkers were set as the reference group. All significance tests were two-tailed, and p-values less than 0.05 were considered statistically significant.

## Results

### Study participants

**Figure 1:**
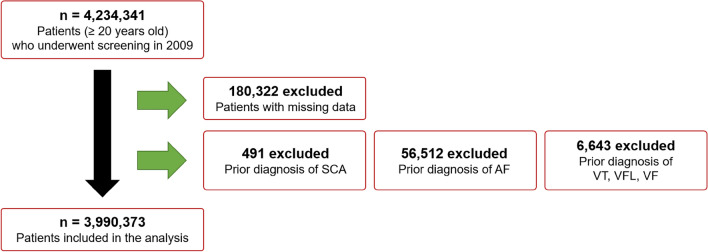
Flow of the study.

A flowchart describing the enrollment of study participants is presented in Fig. 1. After exclusion by age, prior diagnosis, or missing data, 3,990,373 people were included in this study. Participants were classified into non-drinkers, mild drinkers, moderate drinkers, and heavy drinkers, and their baseline demographics are summarized in Table 1. Non-drinkers were more often older, female, and smoked less. In addition, they had lower BMI, waist circumference, and blood pressure; higher low-density lipoprotein (LDL); less regular exercise habits; and higher prevalence of CKD, heart failure, and thyroid disease (Table 1).

**Table 1:** Baseline demographics.

	Non-drinkers 2,043,782 (51.2%)	Mild drinkers 1,167,656 (29.3%)	Moderate drinkers 460,144 (11.5%)	Heavy drinkers 318,845 (8.0%)	*p*-value
Male	704,637 (34.5%)	784,430 (67.2%)	409,477 (89.0%)	299,064 (93.8%)	< 0.001
Age	50.43 ± 14.25	42.75 ± 12.86	43.09 ± 12.18	44.33 ± 12.70	< 0.001
20–29	181,739 (8.9%)	206,240 (17.7%)	68,116 (14.8%)	44,034 (13.8%)	< 0.001
30–39	276,863 (13.6%)	299,547 (25.7%)	122,697 (26.7%)	75,839 (23.8%)	
40–49	507,036 (24.8%)	321,122 (27.5%)	134,599 (29.3%)	93,238 (29.2%)	
50–59	494,874 (24.2%)	202,954 (17.4%)	85,046 (18.5%)	62,790 (19.7%)	
60–69	363,573 (17.8%)	98,202 (8.4%)	37,512 (8.2%)	30,621 (9.6%)	
70–79	189,788 (9.3%)	35,305 (3.0%)	11,074 (2.4%)	11,274 (3.5%)	
80-	29,855 (1.5%)	4,286 (0.4%)	1,100 (0.2%)	1,049 (0.3%)	
BMI	23.59 ± 3.26	23.54 ± 3.17	24.14 ± 3.11	24.42 ± 3.19	< 0.001
Waist circumference	79.11 ± 9.45	80.01 ± 9.42	82.88 ± 8.65	84.12 ± 8.87	< 0.001
Smoking					< 0.001
Non-smoker	1,627,891 (79.7%)	570,788 (48.9%)	109,268 (23.8%)	57,288 (18.0%)	
Ex-smoker	170,903 (8.4%)	218,809 (18.7%)	106,935 (23.2%)	75,201 (23.6%)	
Current smoker	244,934 (12.0%)	378,059 (32.4%)	243,941 (53.0%)	186,356 (58.5%)	
Regular exercise	344,743 (16.9%)	220,002 (18.8%)	92,613 (20.1%)	66,097 (20.7%)	< 0.001
Low income (20%)	394,377 (19.3%)	191,561 (16.4%)	65,443 (14.2%)	45,594 (14.3%)	< 0.001
Diabetes mellitus	192,207 (9.4%)	74,773 (6.4%)	39,407 (8.6%)	34,861 (10.9%)	< 0.001
Non-diabetic	1,426,516 (69.8%)	835,120 (71.5%)	296,377 (64.4%)	189,041 (59.3%)	< 0.001
IFG	425,005 (20.8%)	257,763 (22.1%)	124,360 (27.0%)	94,943 (29.8%)	
New onset DM	50,677 (2.5%)	31,630 (2.7%)	18,987 (4.1%)	16,923 (5.3%)	
DM < 5 years	69,496 (3.4%)	23,395 (2.0%)	11,738 (2.6%)	10,476 (3.3%)	
DM ≥ 5 years	72,034 (3.5%)	19,748 (1.7%)	8,682 (1.9%)	7,462 (2.3%)	
Glucose	96.86 ± 23.74	95.74 ± 21.77	98.86 ± 24.83	101.67 ± 28.35	< 0.001
Hypertension	589,867 (28.9%)	241,490 (20.7%)	122,563 (26.6%)	98,776 (31.0%)	< 0.001
Non-hypertension	744,749 (36.4%)	433,362 (37.1%)	123,459 (26.8%)	72,406 (22.7%)	< 0.001
Pre-hypertension	709,112 (34.7%)	492,804 (42.2%)	214,122 (46.5%)	147,663 (46.3%)	
Hypertension	142,408 (7.0%)	91,362 (7.8%)	53,703 (11.7%)	44,805 (14.1%)	
Hypertension with medication	447,459 (21.9%)	150,128 (12.9%)	68,860 (15.0%)	53,971 (16.9%)	
SBP	121.7 ± 15.43	121.4 ± 14.29	124.95 ± 14.27	126.79 ± 14.71	< 0.001
DBP	75.42 ± 10.01	76.04 ± 9.81	78.58 ± 9.87	79.66 ± 10.10	< 0.001
Dyslipidemia	422,244 (20.7%)	166,041 (14.2%)	73,453 (16.0%)	54,568 (17.1%)	< 0.001
No dyslipidemia	1,621,484 (79.3%)	1,001,615(85.8%)	386,691 (84.0%)	264,277 (82.9%)	< 0.001
Dyslipidemia without medication	178,950 (8.8%)	92,626 (7.9%)	42,317 (9.2%)	30,535 (9.6%)	
Dyslipidemia on medication	243,294 (11.9%)	73,415 (6.3%)	31,136 (6.8%)	24,033 (7.5%)	
Total cholesterol	196.41 ± 42.17	193 ± 39.62	195.8 ± 39.94	196.46 ± 41.38	< 0.001
HDL	56.55 ± 35.39	56.42 ± 29.82	56.19 ± 29.59	57.38 ± 31.12	< 0.001
LDL	123.07 ± 184.78	121.99 ± 254.33	117.38 ± 228.32	112.92 ± 219.84	< 0.001
CKD	163,762 (8.0%)	66,664 (5.7%)	23,098 (5.0%)	14,774 (4.6%)	< 0.001
eGFR	86.32 ± 39.26	88.7 ± 50.87	89.22 ± 49.97	90.35 ± 48.49	< 0.001
Heart failure	47,552 (2.3%)	10,102 (0.9%)	3,540 (0.8%)	2,884 (0.9%)	< 0.001
Thyroid disease	224,762 (11.0%)	68,645 (5.9%)	18,066 (3.9%)	11,701 (3.6%)	< 0.001
Liver cirrhosis	20,028 (1.0%)	7,122 (0.6%)	3,313 (0.7%)	3,539 (1.1%)	< 0.001
Cancer diagnosis	44,144 (2.2%)	9,341 (0.8%)	2,392 (0.5%)	1,594 (0.5%)	< 0.001

CKD: chronic kidney disease; DBP: diastolic blood pressure; DM: diabetes mellitus; eGFR: estimated glomerular filtration rate; HDL: high-density lipoprotein; IFG: impaired fasting glucose; LDL: low-density lipoprotein.

### All-cause mortality

**Figure 2:**
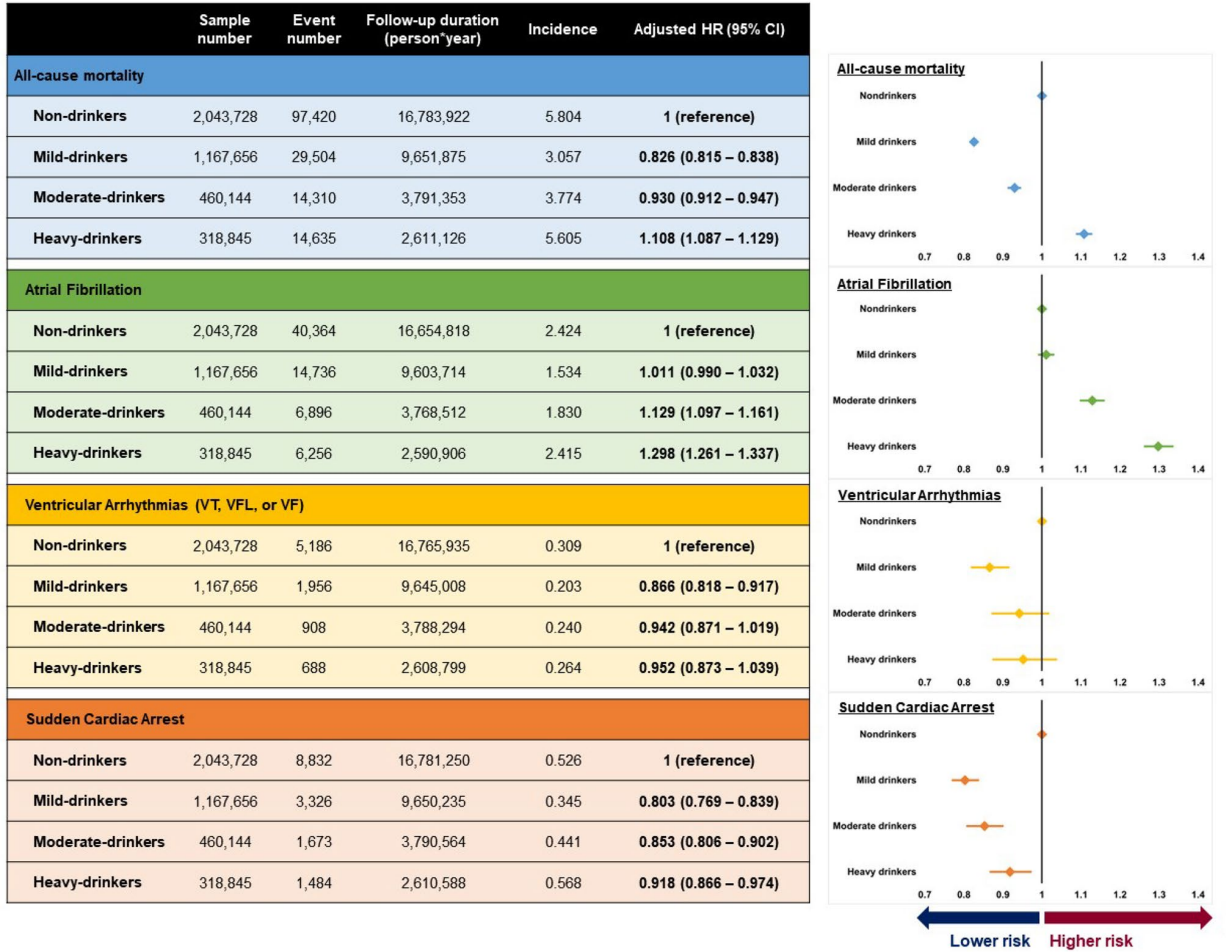
Influence of alcohol consumption Incidence is per 1,000 person*year follow-up. AF: atrial fibrillation; CI: confidence interval; HR: hazard ratio; VA: ventricular arrhythmia; VF: ventricular fibrillation; VFL: ventricular flutter; VT: ventricular tachycardia; SCA: sudden cardiac arrest Alcohol consumption demonstrated different associations with all-cause mortality, atrial fibrillation, ventricular arrhythmias, and sudden cardiac arrest. HRs are adjusted for age, sex, body mass index, smoking status, regular exercise, income level, hypertension, diabetes mellitus, dyslipidemia, chronic kidney disease, heart failure, and thyroid disease.

**Figure 3:**
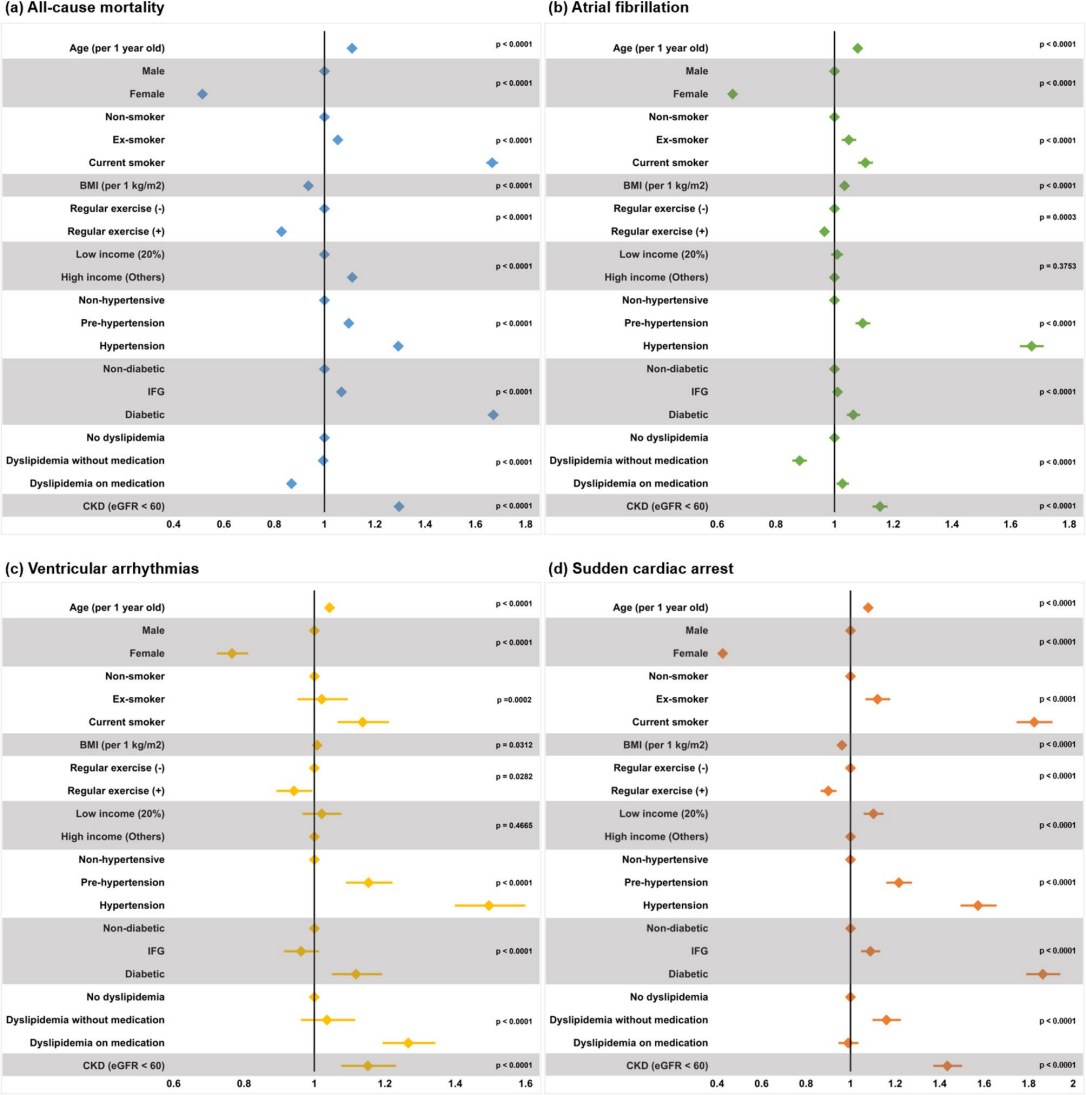
Risk factors for all-cause death, AF, VA, and SCA. Male, non-smoker, no regular exercise, non-hypertensive, non-diabetic, and no dyslipidemia were set as reference values for each set of comparison. For values regarding income, low income (lowest quintile) was set as the reference for all-cause mortality, while high income was set as the reference for the rest of the diseases in interest.

Incidences (per 1,000 person*year follow-up) of death, regardless of the cause, were 5.80, 3.06, 3.77, and 5.61 for non-drinkers, mild drinkers, moderate drinkers, and heavy drinkers, respectively. After adjustment of covariates (multivariate model 4), mild drinkers (HR = 0.83; 95% CI = 0.82–0.84; *p* < 0.01; Table 2 and Fig. 2) and moderate drinkers (HR = 0.93; 95% CI = 0.91–0.95; *p* < 0.01; Table 2 and Fig. 2) showed significantly decreased risks of all-cause mortality. On the other hand, heavy drinkers had a 10.8% increased risk of all-cause death (HR = 1.11; 95% CI = 1.09–1.13; *p* < 0.01; Table 2 and Fig. 2). The impact of various covariates on all-cause death is depicted in Fig. 3 (multivariate model 3). Old age, male sex, smoking, lack of regular exercise, high income, hypertension, diabetes mellitus, and CKD were major risk factors for all-cause mortality. Presence of dyslipidemia did not increase the risk, and high BMI was associated with lower risk (Fig. 3a).

**Table 2:** Alcohol and risk of all-cause mortality, AF, VA, and SCA.

	Sample number	Event number	Person*year	Univariate	Model 1	Model 2	Model 3	Model 4	Model 5
All-cause mortality									
Non-drinker	2,043,728	97,420	16,783,922	1 (reference)	1 (reference)	1 (reference)	1 (reference)	1 (reference)	1 (reference)
Mild drinker	1,167,656	29,504	9,651,875	0.528 (0.521–0.535)	0.851 (0.839–0.862)	0.822 (0.810–0.833)	0.824 (0.812–0.835)	0.826 (0.815–0.838)	0.836 (0.825–0.847)
Moderate drinker	460,144	14,310	3,791,353	0.653 (0.641–0.664)	1.018 (0.999–1.037)	0.938 (0.920–0.956)	0.927 (0.909–0.944)	0.930 (0.912–0.947)	0.932 (0.915–0.950)
Heavy drinker	318,845	14,635	2,611,126	0.969 (0.952–0.986)	1.249 (1.226–1.272)	1.124 (1.103–1.145)	1.104 (1.083–1.125)	1.108 (1.087–1.129)	1.104 (1.084–1.124)
Atrial fibrillation									
Non-drinker	2,043,728	40,364	16,654,818	1 (reference)	1 (reference)	1 (reference)	1 (reference)	1 (reference)	1 (reference)
Mild drinker	1,167,656	14,736	9,603,714	0.634 (0.622–0.646)	1.009 (0.989–1.030)	1.006 (0.985–1.027)	1.003 (0.982–1.024)	1.011 (0.990–1.032)	1.014 (0.994–1.035)
Moderate drinker	460,144	6,896	3,768,512	0.756 (0.737–0.776)	1.168 (1.136–1.200)	1.138 (1.107–1.171)	1.118 (1.087–1.150)	1.129 (1.097–1.161)	1.135 (1.103–1.167)
Heavy drinker	318,845	6,256	2,590,906	0.998 (0.972–1.025)	1.342 (1.304–1.381)	1.306 (1.269–1.345)	1.286 (1.248–1.324)	1.298 (1.261–1.337)	1.298 (1.260–1.336)
Ventricular arrhythmia									
Non-drinker	2,043,728	5,186	16,765,935	1 (reference)	1 (reference)	1 (reference)	1 (reference)	1 (reference)	1 (reference)
Mild drinker	1,167,656	1,956	9,645,008	0.656 (0.623–0.691)	0.864 (0.816–0.913)	0.858 (0.810–0.908)	0.859 (0.812–0.910)	0.866 (0.818–0.917)	0.867 (0.819–0.917)
Moderate drinker	460,144	908	3,788,294	0.776 (0.723–0.832)	0.973 (0.901–1.050)	0.943 (0.872–1.019)	0.933 (0.863–1.009)	0.942 (0.871–1.019)	0.943 (0.872–1.019)
Heavy drinker	318,845	688	2,608,799	0.854 (0.788–0.924)	0.988 (0.908–1.076)	0.952 (0.873–1.038)	0.942 (0.864–1.028)	0.952 (0.873–1.039)	0.952 (0.873–1.039)
Sudden cardiac arrest									
Non-drinker	2,043,728	8,832	16,781,250	1 (reference)	1 (reference)	1 (reference)	1 (reference)	1 (reference)	1 (reference)
Mild drinker	1,167,656	3,326	9,650,235	0.656 (0.631–0.683)	0.840 (0.805–0.876)	0.799 (0.766–0.834)	0.800 (0.767–0.836)	0.803 (0.769–0.839)	0.815 (0.781–0.851)
Moderate drinker	460,144	1,673	3,790,564	0.841 (0.798–0.886)	0.970 (0.918–1.026)	0.868 (0.820–0.918)	0.850 (0.803–0.899)	0.853 (0.806–0.902)	0.849 (0.803–0.899)
Heavy drinker	318,845	1,484	2,610,588	1.083 (1.025–1.144)	1.073 (1.012–1.137)	0.941 (0.888–0.999)	0.915 (0.862–0.971)	0.918 (0.866–0.974)	0.928 (0.875–0.984)

Values are expressed as hazard ratio with its 95% confidence interval.

Multivariate model 1: adjusted for age and sex.

Multivariate model 2: model 1 plus body mass index, smoking status, regular exercise habit, and income level.

Multivariate model 3: model 2 plus hypertension, diabetes mellitus, dyslipidemia, and chronic kidney disease.

Multivariate model 4: model 3 plus history of heart failure and thyroid disease.

Multivariate model 5: model 4 plus history of liver cirrhosis and cancer diagnosis.

### Atrial fibrillation

We observed a considerably higher incidence of AF in people who drink large amounts of alcohol. The incidence of AF was 2.42, 1.53, 1.83, and 2.42 for non-drinkers, mild drinkers, moderate drinkers, and heavy drinkers, respectively. After adjustment of covariates (multivariate model 4), moderate drinking (HR = 1.13; 95% CI = 1.10–1.16; *p* < 0.01; Table 2 and Fig. 2) and heavy drinking (HR = 1.30; 95% CI = 1.26–1.34; *p* < 0.01; Table 2 and Fig. 2) were associated with significantly increased risk of AF. Mild drinkers had a similar risk of AF compared with non-drinkers (HR = 1.01; 95% CI = 0.99–1.03; p = 0.30; Table 2 and Fig. 2). Other factors associated with increased risk of AF were old age, male sex, smoking, high BMI, lack of regular exercise, hypertension, diabetes mellitus, and CKD. Dyslipidemia did not show any consistent association with AF (Fig. 3b).

### Ventricular arrhythmias

The association between alcohol and VAs was different from that of AF. In comparison with non-drinkers, mild drinkers showed significantly lower risk of VA (HR = 0.87; 95% CI = 0.82–0.92; *p* < 0.01; Table 2 and Fig. 2). Moderate drinking and heavy drinking were associated with numerically, but not statistically, lower risk of VA (Table 2 and Fig. 2). Risk factors associated with increased risk of VA were old age, male sex, current smoker, lack of regular exercise, hypertension, diabetes mellitus, dyslipidemia, and CKD (Fig. 3c).

### Sudden cardiac arrest

The incidences were 0.53, 0.35, 0.44, and 0.57, for non-drinkers, mild drinkers, moderate drinkers, and heavy drinkers, respectively. Multivariate analysis demonstrated that alcohol consumption was associated with lower risk of SCA regardless of the amount: (i) Mild drinkers: HR = 0.80; 95% CI = 0.77–0.84; *p* < 0.01, (ii) Moderate drinkers: HR = 0.85; 95% CI = 0.81–0.90; *p* < 0.01, and (iii) Heavy drinkers: HR = 0.92; 95% CI = 0.87–0.97; p = 0.01 (Table 2 and Fig. 2). Old age, male sex, smoking, lower BMI, lack of regular exercise, low income, hypertension, diabetes mellitus, and CKD were risk factors significantly associated with SCA. Dyslipidemia showed an inconsistent association (Fig. 3d).

### Heart failure admission

Mild drinkers showed 15.3% decreased risk of heart failure admission compared with non-drinkers (HR = 0.85; 95% CI = 0.83–0.86; *p* < 0.01). Moderate drinking was also associated with lower risk of heart failure (HR = 0.88; 95% CI = 0.86–0.90; *p* < 0.01). However, heavy drinking was associated with similar risk of heart failure admission compared with non-drinking (HR = 1.00; 95% CI = 0.98–1.03).

### Subgroup analysis

The interaction between alcohol consumption and sex on each outcome was evaluated (Table 3). Increased risk of AF in drinkers was more pronounced in males. However, females were more vulnerable to alcohol in terms of all-cause death. No remarkable interactions were observed between alcohol and sex in ventricular arrhythmias and SCA. Age also did not show any meaningful interactions with alcohol consumption in all outcomes.

**Table 3:** Subgroup analysis.

Male						
	*n*	SCA	VT / VFL / VF	AF	All-cause death	HF admission
Non-drinker	704,637	1 (reference)	1 (reference)	1 (reference)	1 (reference)	1 (reference)
Mild drinker	784,430	0.772 (0.736–0.810)	0.860 (0.803–0.921)	1.000 (0.976–1.025)	0.798 (0.785–0.810)	0.880 (0.870–0.890)
Moderate drinker	409,477	0.823 (0.776–0.872)	0.918 (0.844–0.998)	1.123 (1.090–1.156)	0.894 (0.876–0.912)	0.900 (0.887–0.913)
Heavy drinker	299,064	0.910 (0.858–0.967)	1.000 (0.915–1.048)	1.271 (1.233–1.310)	1.074 (1.054–1.095)	0.959 (0.945–0.973)
Female						
	*n*	SCA	VT / VFL / VF	AF	All-cause death	HF admission
Non-drinker	1,339,091	1 (reference)	1 (reference)	1 (reference)	1 (reference)	1 (reference)
Mild drinker	383,226	1.042 (0.945–1.150)	0.810 (0.733–0.896)	1.010 (0.972–1.051)	1.009 (0.987–1.041)	0.948 (0.934–0.963)
Moderate drinker	50,667	1.202 (0.946–1.527)	1.013 (0.806–1.273)	1.086 (0.980–1.204)	1.335 (1.237–1.440)	1.024 (0.986–1.063)
Heavy drinker	19,781	0.951 (0.634–1.427)	1.063 (0.753–1.499)	1.174 (1.007–1.370)	1.354 (1.206–1.519)	1.071 (1.012–1.133)
Young age (< 65 years)						
	*n*	SCA	VT / VFL / VF	AF	All-cause death	HF admission
Non-drinker	1,670,748	1 (reference)	1 (reference)	1 (reference)	1 (reference)	1 (reference)
Mild drinker	1,090,707	0.800 (0.755–0.848)	0.850 (0.796–0.907)	0.982 (0.956–1.009)	0.857 (0.839–0.875)	0.910 (0.900–0.920)
Moderate drinker	434,422	0.845 (0.787–0.907)	0.885 (0.810–0.966)	1.097 (1.059–1.135)	0.960 (0.935–0.985)	0.919 (0.906–0.933)
Heavy drinker	295,059	0.928 (0.861–1.000)	1.018 (0.926–1.119)	1.261 (1.216–1.309)	1.192 (1.161–1.225)	0.983 (0.967–0.999)
Old age (≥ 65 years)						
	*n*	SCA	VT / VFL / VF	AF	All-cause death	HF admission
Non-drinker	372,980	1 (reference)	1 (reference)	1 (reference)	1 (reference)	1 (reference)
Mild drinker	76,949	0.846 (0.792–0.902)	0.860 (0.767–0.964)	1.038 (1.005–1.072)	0.833 (0.817–0.849)	0.928 (0.913–0.944)
Moderate drinker	25,722	0.839 (0.760–0.926)	1.082 (0.919–1.274)	1.140 (1.086–1.197)	0.906 (0.881–0.932)	0.925 (0.900–0.950)
Heavy drinker	23,786	0.901 (0.817–0.993)	0.874 (0.726–1.052)	1.223 (1.164–1.284)	1.018 (0.991–1.047)	0.932 (0.906–0.958)

Values are expressed as hazard ratio with its 95% confidence interval.

AF: atrial fibrillation; VT: ventricular tachycardia; VF: ventricular fibrillation; VFL: ventricular flutter; SCA: sudden cardiac arrest.

Hazard ratios are adjusted for age, sex, body mass index, smoking status, regular exercise habit, income level, hypertension, diabetes mellitus, dyslipidemia, chronic kidney disease, heart failure, and thyroid disease.

## Discussion

In this study, we analyzed the relationship between the amount of alcohol intake and all-cause mortality, AF, VA (VT, VFL, or VF), and SCA. The principal findings of this study are as follows. (i) The association between alcohol consumption and risk of all-cause mortality is U-shaped. Compared to non-drinkers, mild to moderate drinkers had lower risk, while heavy drinkers had higher risk. (ii) Moderate to heavy drinkers had a significantly increased risk of AF compared with non-drinkers. (iii) Alcohol intake, regardless of amount, was associated with lower risk of SCA. (iv) Mild drinkers had lower risk of VA compared with non-drinkers. The strong points of our study are a large sample size, adjustment of various covariates, and analysis of all-cause mortality, AF, VA, and SCA in the same cohort. In this way, we were able to reveal the different influence of alcohol on AF vs. VA and SCA.

### Alcohol and cardiac arrhythmias

The association between alcohol and AF is demonstrated in multiple prior studies^14,15^. The lower risk of all-cause mortality in mild to moderate drinkers was also observed in other observational studies^12^. Our study showed similar results in a different ethnic group with a larger sample size utilizing nationwide health insurance data.

It has long been discussed that alcohol intake and the risk of VA and SCA may have a specific relationship, but this has not been clarified^19^. Prior studies of VA with limited sample sizes showed inconsistent results^11,20,21^. One study of patients with myocardial infarction who did not receive thrombolytic therapy stated that frequency of drinking was not associated with an increased risk of VA^20^, while another study of patients without cardiovascular disease concluded that heavy consumption of alcohol is a major risk factor for VA^21^. Using the UK Biobank cohort, a recent study gathered a much larger and more generalized population^11^. The study reported a U-shaped relationship such that those who drank less than 208 g of alcohol per week (in the current study, mild and moderate drinkers) had less risk of SCA, while the conclusion for VAs was not statistically significant^11^. In our study, not only mild to moderate drinkers, but also heavy drinkers had a significantly lower risk of SCA. For VAs, mild drinkers had a significantly lower risk compared with non-drinkers. Moderate and heavy drinkers had numerically, but not statistically significant, lower risk of VAs. To our knowledge, this is the study with the largest sample size (*n* = 3,990,373) to evaluate the associations between alcohol and various cardiac disorders simultaneously.

### Biological mechanism

Alcohol consumption can cause pathophysiological changes to the heart, such as alterations in the atrial effective refractory period or the autonomic nervous system^15,22,23^. Elevation in blood pressure, obesity, and direct toxic effects on cardiomyocytes are other explanations for the association between alcohol consumption and increased risk of new-onset AF^15,24,25^.

Ventricular arrhythmias can be initiated by various factors, but the main cause is acute ischemia of the heart or pre-existing ischemia-related scar^26^. Coronary artery disease is also a main cause of SCA. It is widely perceived that a moderate alcohol intake can have a protective effect on coronary artery disease^27,28^, and one systematic review reported that the risk significantly decreases when consuming more than 30 g of alcohol per day^28^, a criterion classified as heavy drinking (> 210 g per week) in the current study. This effect can be explained by changes in cholesterol levels and coagulation factors, both related to atherosclerosis and thrombogenesis in the coronary arteries. Intake of alcohol is associated with higher synthesis and slower decrease in high-density lipoprotein (HDL) level, thereby maintaining an upregulated level of HDL^29,30^. Furthermore, our study revealed an inverse linear association between amount of alcohol intake and LDL level (Table 1). High HDL and low LDL can have a prohibitory effect on the underlying inflammatory process of atherosclerosis; as a result, the risk of coronary artery disease might decrease as well as the risk of VAs and SCA. Although investigated at a cellular level, alcohol is also proven to have an effect on upregulating expression and synthesis of fibrinolytic proteins such as tissue plasminogen activator, which can prohibit the formation of thrombi^31,32^.

### Clinical implications

Ventricular arrhythmia is strongly connected to the risk of SCA, a major socioeconomic burden. Due to a narrow therapeutic window, most SCA events have low chance of neurologically intact survival^33^. Atrial fibrillation is another major burden for the general population since its prevalence is up to 2%^34^. Primary prevention of both diseases will have a significant impact on public health. Although alcohol consumption was associated with significantly lower risk of SCA, the risk of new-onset AF was significantly increased. Furthermore, significantly increased risk of all-cause death was observed in the heavy-drinker group. Mild to moderate intake of alcohol might have benefits in terms of prevention of SCA and all-cause death. However, due to potential bias originating from retrospective analysis, heterogeneous effect of alcohol on individuals, and non-cardiac hazardous effects such as carcinogenesis, we cannot conclude whether mild to moderate intake of alcohol will be beneficial for public health^35,36^. Regarding the risk of new-onset AF, cessation or reducing alcohol intake might be helpful for the prevention in the general population.

### Limitations

This study was based on the database of K-NHIS, which exclusively comprises East Asian ethnicity, so it is necessary to consider ethnic differences when applying the result to other ethnic groups. Also, not just one, but multiple measurements of alcohol consumption habits should be considered in future studies since changes in alcohol intake habits can have independent association with cardiac arrhythmia and SCA. Since this study was based on a claim database, unmeasured confounders and coding inaccuracies might exist. Despite vigorous efforts to adjust various confounders in the multivariable models, residual confounders can exist. Diagnosis of AF, VT, and VF was based on reports of relevant ICD-10 codes and electrocardiography documentation was not possible.

## Conclusion

New-onset AF and SCA (including VAs) showed different directions of association with alcohol intake. Opposite association with alcohol intake suggests that AF and SCA (including VAs) have different pathophysiology. All-cause death was lower in mild to moderate drinkers but higher in heavy drinkers. Since the risk of SCA in heavy drinkers was higher than in mild and moderate drinkers, despite being lower than in non-drinkers, heavy drinking should be avoided.

### Supplementary Information

Supplementary Tables.

## Data Availability

All data generated or analyzed during this study are included in this article. Further enquiries can be directed to the corresponding author. The raw data cannot be shared due to legal regulation of Republic of Korea and policy of the K-NHIS.

## Abbreviations

AF: Atrial fibrillation
BMI: Body mass index
CI: Confidence interval
CKD: Chronic kidney disease
DM: Diabetes mellitus
eGFR: Estimated glomerular filtration rate
HDL: High-density lipoprotein
HR: Hazard ratio
ICD: International classification of disease
K-NHIS: Korean National Health Insurance Service
LDL: Low-density lipoprotein
SCA: Sudden cardiac arrest
VA: Ventricular arrhythmia
VF: Ventricular fibrillation
VFL: Ventricular flutter
VT: Ventricular tachycardia
