# Supplementary material for: Alcohol and the risk of all-cause death, atrial fibrillation, ventricular arrhythmia, and sudden cardiac arrest
Source: Sci Rep. 2024 Feb 29;14:5053. doi: 10.1038/s41598-024-55434-6 (PMC10904378; doi:10.1038/s41598-024-55434-6)
Supplement: Supplementary file 1 — Supplementary Tables. [file 41598_2024_55434_MOESM1_ESM.docx]

**Alcohol and the Risk of All-cause Death, Atrial Fibrillation, Ventricular Arrhythmia, and Sudden Cardiac Arrest**

Yun Gi Kim,^1#^ Dong Yun Kim,^2#^ Seung-Young Roh,^3^ Joo Hee Jeong,^1^ Hyoung Seok Lee,^1^ Kyongjin Min,^1^ Yun Young Choi,^1^ Kyung-Do Han,^4^ Jaemin Shim,^1^ Jong-Il Choi,^1^* and Young-Hoon Kim^1^

^1^Division of Cardiology, Department of Internal Medicine, Korea University College of Medicine and Korea University Anam Hospital, Seoul, Republic of Korea

^2^Korea University College of Medicine, Seoul, Republic of Korea

^3^Division of Cardiology, Department of Internal Medicine, Korea University College of Medicine and Korea University Guro Hospital, Seoul, Republic of Korea

^4^Department of Statistics and Actuarial Science, Soongsil University, Seoul, Republic of Korea

*Address for correspondence: Jong-Il Choi, MD, PhD, MHSc

^1^Division of Cardiology, Department of Internal Medicine, Korea University College of Medicine and Korea University Anam Hospital, Seoul, Republic of Korea

73 Goryeodae-ro, Seongbuk-gu, Seoul 02841, Republic of Korea

Tel: 82-2-920-5445 / Fax: 82-2-927-1478 / E-mail: [jongilchoi@korea.ac.kr](mailto:jongilchoi@korea.ac.kr)

^#^The first two authors contributed equally to this work.

**Running title:** Alcohol, arrhythmia and sudden cardiac arrest

**Disclosure:** The authors have nothing to disclose.

**Total word count:** 5,762

**Supplementary Table S1.** Classification of patients by total amount of alcohol intake per week.

| Classification | Total amount of alcohol intake per week (g) |
| --- | --- |
| Non-drinker | 0 |
| Mild drinker | >0, <105 |
| Moderate drinker | ≥ 105, <210 |
| Heavy drinker | ≥ 210 |

**Supplementary Table S2.** ICD-10 codes for diagnosis.

| **Diseases** | **ICD-10 Codes** |
| --- | --- |
| **Atrial fibrillation** | I48 (all sub-codes) |
| **Ventricular arrhythmia composite** | I47.2, I49.0 |
| Ventricular tachycardia | I47.2 (all sub-codes) |
| Ventricular flutter and ventricular fibrillation | I49.0 |
| **Sudden cardiac arrest** | I46.0, I46.1, I46.9, I49.0, R96.0, R96.1 |
| Cardiac arrest with successful resuscitation | I46.0 |
| Sudden cardiac arrest | I46.1 |
| Cardiac arrest, cause unspecified | I46.9 |
| Ventricular fibrillation and flutter | I49.0 |
| Instantaneous death | R96.0 |
| Death occurring less than 24 hours from symptom onset | R96.1 |
| **Hypertension** | I10 – I13, I15 (all sub-codes) |
| **Diabetes mellitus (Type 2 Diabetes)** | E11 – E14 (all sub-codes) |
| **Dyslipidemia** | I78 (all sub-codes) |
| **Chronic kidney disease** | Based on the creatinine level checked during national health screening |
| **Heart failure** | I50 (all sub-codes) |
| **Thyroid disease (Hypo- or Hyperthyroidism)** | E03 (all sub-codes), E05 (all sub-codes) |
| **Liver cirrhosis** | K70.3, K74.6 |
| **Cancer** | All ICD-10 codes that starts with initial “C” accompanied by special discount by the K-NHIS* |

ICD-10: International Classification of Diseases, tenth edition; K-NHIS: National Health Insurance Service of the Republic of Korea.

*The K-NHIS offers 95% discount service to cancer patients registered by the duty physician. The approval is based on strict examination by the K-NHIS ensuring the robustness of our definition of cancer.

**Supplementary Table S3.** Definitions of levels of smoking, diabetes mellitus, hypertension, and dyslipidemia used in this study

|  | Definition |
| --- | --- |
| **Smoking** | |
| Non-smoker | <100 cigarettes in the lifetime |
| Ex-smoker | ≥100 cigarettes in the lifetime but did not smoke within 1 month of health check-up in 2009 |
| Current smoker | ≥100 cigarettes in the lifetime and continued smoking within 1 month of health check-up in 2009 |
| **Diabetes mellitus** | |
| Non-diabetic | FBG < 100 mg/dl and no diagnostic code for diabetes mellitus |
| IFG | 100 ≤ FBG < 126 no diagnostic code for diabetes mellitus |
| Diabetic | FBG ≥ 126 and diagnostic code for diabetes mellitus |
| **Hypertension** | |
| Non-hypertensive | SBP < 120, DBP < 80, and no diagnostic code for hypertension |
| Pre-hypertension | 120 ≤ SBP < 140 or 80 ≤ DBP < 90, no diagnostic code for hypertension |
| Hypertension | SBP ≥ 140 or DBP ≥ 90 or diagnostic codes for hypertension |
| **Dyslipidemia** | |
| No dyslipidemia | Total cholesterol < 240 |
| Dyslipidemia without medication | Total cholesterol ≥ 240 |
| Dyslipidemia on medication | Total cholesterol ≥ 240 with medication |

DBP: Diastolic blood pressure, mmHg; FBG: Fasting blood glucose, mg/dL; IFG: Impaired fasting glucose; SBP: Systolic blood pressure, mmHg.
